# Ionizing radiation increases the endothelial permeability and the transendothelial migration of tumor cells through ADAM10-activation and subsequent degradation of VE-cadherin

**DOI:** 10.1186/s12885-019-6219-7

**Published:** 2019-10-16

**Authors:** Pascaline Nguemgo Kouam, Günther A. Rezniczek, Irenäus A. Adamietz, Helmut Bühler

**Affiliations:** 1 Institute for Molecular Oncology, Radio-Biology and Experimental Radiotherapy, Ruhr-Universität Bochum, Medical Research Center, Marien Hospital Herne, Hölkeskampring 40, 44265 Herne, Germany; 2 Department of Radiotherapy and Radio-Oncology, Ruhr-Universität Bochum, Medical Research Center, Marien Hospital Herne, Hölkeskampring 40, 44265 Herne, Germany; 3 Department of Obstetrics and Gynecology, Ruhr-Universität Bochum, Medical Research Center, Marien Hospital Herne, Hölkeskampring 40, 44265 Herne, Germany

**Keywords:** Irradiation, Endothelium, VE-cadherin, Metalloproteinase, Permeability

## Abstract

**Background:**

We analyzed the changes in permeability of endothelial cell layers after photon irradiation, with a focus on the metalloproteases ADAM10 and ADAM17, and on VE-cadherin, components crucial for the integrity of endothelial intercellular junctions, and their roles in the transmigration of cancer cells through endothelial cell monolayers.

**Methods:**

Primary HUVEC were irradiated with 2 or 4 Gy photons at a dose rate of 5 Gy/min. The permeability of an irradiated endothelial monolayer for macromolecules and tumor cells was analyzed in the presence or absence of the ADAM10/17 inhibitors GI254023X and GW280264X. Expression of ADAM10, ADAM17 and VE-Cadherin in endothelial cells was quantified by immunoblotting and qRT. VE-Cadherin was additionally analyzed by immunofluorescence microscopy and ELISA.

**Results:**

Ionizing radiation increased the permeability of endothelial monolayers and the transendothelial migration of tumor cells. This was effectively blocked by a selective inhibition (GI254023X) of ADAM10. Irradiation increased both, the expression and activity of ADAM10, which led to increased degradation of VE-cadherin, but also led to higher rates of VE-cadherin internalization. Increased degradation of VE-cadherin was also observed when endothelial monolayers were exposed to tumor-cell conditioned medium, similar to when exposed to recombinant VEGF.

**Conclusions:**

Our results suggest a mechanism of irradiation-induced increased permeability and transendothelial migration of tumor cells based on the activation of ADAM10 and the subsequent change of endothelial permeability through the degradation and internalization of VE-cadherin.

## Background

Radiotherapy is a principal treatment method in clinical oncology, being an effective means of local tumor control and having curative potential for many cancer types. However, there were various observations in the earliest stages of radiation oncology that ineffective irradiation of solid tumors could ultimately result in the enhancement of metastasis. Several clinical studies have revealed that patients with local failure after radiation therapy were more susceptible to develop distant metastasis than those with local tumor control [1–3]. However, how ionizing radiation may be involved in the molecular mechanisms leading to tumor dissemination and metastasis formation is not well understood.

During the metastatic cascade, a single cancer cell or a cluster of cancer cells first detaches from the primary tumor, then invades the basement membrane and breaks through an endothelial cell layer to enter into a lymphatic or blood vessel (intravasation). Tumor cells are then circulating until they arrive at a (distant) site where they perform extravasation [4, 5]. This process depends on complex interactions between cancer cells and the endothelial cell layer lining the vessel and can be divided into three main steps: rolling, adhesion, and transmigration [4, 6]. In this last step, cancer cell have to overcome the vascular endothelial (VE) barrier, which is formed by tight endothelial adherence junctions and VE-cadherin as their major component [7, 8]. Thus, VE-cadherin is an essential determinant of the vascular integrity [9, 10] and plays an important role in controlling endothelial permeability [11], leukocyte transmigration, and angiogenesis [12]. Recent studies have shown that VE-cadherin is a substrate of the ADAM10 (a disintegrin and metalloproteinase 10) and that its activation leads to an increase in endothelial permeability [13].

We hypothesized that degradation of VE-cadherin through ADAM10 is a relevant mechanism contributing to the invasiveness of cancer cells that might be modulated by ionizing irradiation. Therefore, we analyzed changes in the permeability of endothelial cell layers for tumor cells after irradiation, with a particular focus on the transmigration process, by measuring the expression levels of VE-cadherin and modulating, through inhibitors, the activity of ADAM metalloproteases.

## Methods

### Cell culture

The breast cancer cell line MDA-MB-231 and the glioblastoma cell line U-373 MG were obtained from the American Type Culture Collection (ATCC, Manassas, VA, USA). Cells were cultured in Dulbecco’s modified Eagle’s medium (DMEM; #FG0445, Biochrom, Berlin, Germany), supplemented with 10% fetal calf serum (FCS, #S0115/1318D, Biochrom), and penicillin/streptomycin (100 U/ml and 100 μg/ml, respectively; #A2213, Biochrom) (M10), at 37 °C and 5% CO_2_. Primary human umbilical vein endothelial cells (HUVEC; #C-12206, PromoCell, Heidelberg, Germany) were cultured in Endopan medium without VEGF (#P0a-0010 K, PAN-Biotech, Aidenbach, Germany) at 37 °C and 5% CO_2_ for at most six passages.

### Reagents and antibodies

The following chemicals were used: ADAM10 inhibitor (GI254023X; #SML0789, Sigma-Aldrich, Taufkirchen, Germany); ADAM10/17 inhibitor (GW280264X; #AOB3632, Aobious Inc., Hopkinton, MA, USA); human VEGF-A (#V4512, Sigma-Aldrich); TNFα (#H8916, Sigma-Aldrich); protease activator APMA (P-aminophenylmercuric acetate; #A9563, Sigma-Aldrich); γ-secretase inhibitor (flurbiprofen [(R)-251,543.40–9]; #BG0610, BioTrend, Cologne, Germany).

For Western blotting, primary antibodies reactive with the following antigens were used: P-β-catenin (Tyr142; diluted 1:500; #ab27798, abcam, Cambridge, UK); P-VEGF-R2 (Tyr1214; 1:1000, #AF1766, R&D Systems, Wiesbaden, Germany); VE-cadherin (BV9; 1:500; #sc-52,751, Santa Cruz Biotechnology, Heidelberg, Germany); VE-cadherin (1:1000; #2158S); ADAM10 (1:500–1:1000; #14194S); ADAM17 (1:1000; #3976S), β-catenin (1:1000; #9587S); VEGF-R2 (1:1000; #9698S); P-VEGF-R2 (Tyr1175; 1:1000; #2478S, all from Cell Signaling Technology, Frankfurt, Germany); and β-actin-POD (1:25,000; #A3854, Sigma-Aldrich). HRP-conjugated secondary antibodies were from Cell Signaling Technology.

For immunofluorescence microscopy, the following antibodies were used: anti-VE-cadherin (1:50; #2158S); anti-mouse IgG (H + L), Alexa Fluor 555 conjugate (1:1500; #4409); and anti-rabbit IgG (H + L), Alexa Fluor 488 conjugate (1:1500; #4412) (all from Cell Signaling Technology).

### Irradiation

Cells were irradiated with doses of 2 to 4 Gy at a rate of 5 Gy/minute using a commercial linear accelerator (Synergy S, Elekta, Hamburg, Germany), at room temperature. The culture medium was changed 30 min prior to irradiation.

To obtain conditioned medium, 10^6^ tumor cells were seeded in 9-cm^2^-dishes, and grown overnight in M10. Before irradiation as described above, cells were rinsed twice with PBS and covered with 1 ml fresh M10. After irradiation, cells were incubated for 24 h at 37 °C and 5% CO_2_ before the supernatant was harvested. Conditioned medium was filtered (to remove cell debris) and stored at − 20 °C until use. Non-irradiated control samples were treated identically (transport to the accelerator, incubation).

### Permeability assay

The permeability assay (In vitro vascular permeability assay kit; #ECM644, Merck, Darmstadt, Germany) was performed following the manufacturer’s instructions. In brief, 400,000 primary HUVECs were seeded into collagen-coated inserts and cultivated for 48 to 72 h at 37 °C and 5% CO_2._ To determine the permeability of the monolayer, a FITC-Dextran solution (included in the kit) was added to the cells. After incubation for up to 120 min, 100 μl from the lower chamber were transferred into a black 96-well plate and fluorescence (excitation at 485 nm, emission at 535 nm) was measurement in a TECAN Infinite M200 (Tecan, Männedorf, Switzerland).

### Transmigration assay

The transmigration assay (QCMTM tumor cell transendothelial migration assay colorimetric kit; #ECM558, Merck) was performed as suggested by the manufacturer. Here, 250,000 primary HUVECs were seeded into a fibronectin-coated insert and cultured for 96 h at 37 °C and 5% CO_2_ before 100,000 tumor cells were put on top of the monolayer. The transmigration of tumor cells was quantified after 24 h by measuring the absorbance at 570 nm in a TECAN reader.

### Protein isolation and Immunoblot analysis

To isolate proteins from monolayer cell cultures, medium was aspirated, cells were washed with PBS, and subsequently lysed in 1x Roti-Load sample buffer (Carl Roth, Karlsruhe, Germany) with additional homogenization using an ultrasonic probe (Misonix, Farmingdale, NY, USA). Lysates were incubated at 90 °C for 5 min and cleared by centrifugation (1 min, 10,000 g). 15 μl of the protein lysates were separated using SDS-8%-PAGE and blotted onto nitrocellulose membranes (Schleicher & Schüll, Dassel, Germany) in a tank blot unit (Mini-PROTEAN II, BioRad, Hercules, CA, USA). After blocking with a 3% BSA solution, membranes were incubated with primary antibodies, washed, and incubated with HRP-conjugated secondary antibodies. After adding Lumi-Light plus Western Blotting Substrate (Roche Diagnostics, Mannheim, Germany), chemiluminescence was recorded using a ChemiDoc MP system and evaluated using the Image Lab program (both from Bio-Rad).

### Immunofluorescence microscopy

HUVECs were seeded onto glass coverslips and cultured at 37 °C and 5% CO_2_ until confluence. Irradiated or treated cells were first fixed with 4% formaldehyde for 15 min at room temperature, then washed three times with PBS, and finally permeabilized for 10 min with − 20 °C-cold methanol. After removing methanol, coverslips were blocked for 60 min at room temperature in a moist chamber. Incubation with primary antibody was performed overnight at 4 °C. Coverslips were then washed three times for 5 min in the wash buffer and then incubated with the conjugated secondary antibodies for 2 h at room temperature in a moist chamber. Finally, nuclei were stained for 5 min with a 1-μg/ml-Hoechst 33342 solution. The blocking solution, the formaldehyde, the wash buffer, and the dilution buffer for the antibodies were from a kit (#12727, Cell Signaling Technology). Imaging and data analysis were performed using a NIKON ECLIPSE 50i microscope and NIS-Elements AR Microscope Image Software (Nikon, Düsseldorf, Germany).

### Quantitative PCR

Total RNA was isolated from cultured cells using the Total RNA Isolation NucleoSpin RNA II kit (Macherey-Nagel, Düren, Germany). cDNA was reverse transcribed from 1 μg RNA (QuantiTect Reverse Transcription kit; Qiagen, Hilden, Germany). 2 μl of the cDNA (diluted 1:15) were used in PCR reactions consisting of 5 μl 2x QuantiTect SYBR Green buffer (Qiagen) and 3 μl primer mix. Primers used were VE-cadherin (Hs_CDH5_5_SG; #QT00013244), ADAM10 (Hs_ADAM10_1_SG; #QT00032641), ADAM17 (Hs_ADAM17_1_SG; #QT00055580), and GAPDH (Hs_GAPDH_2_SG; #QT01192646) (all from Qiagen). Samples were run in triplicates on a 7900HT real-time PCR system (Applied Biosystems, Darmstadt, Germany). Data were analyzed using the SDS software (Applied Biosystems). In each sample, expression levels were normalized using the mRNA expression of the housekeeping gene GAPDH.

### Quantification of soluble VE-cadherin and VEGF

The hVE-cadherin Quantikine kit (#DCADV0, R&D Systems) was used to measure soluble VE-cadherin in the culture medium and the hVEGF Quantikine kit (#DVE00, R&D Systems) was used to quantify secreted VEGF in the culture medium of tumor cells. These enzyme-linked immunosorbent assay (ELISA) was performed according to the kit instructions.

### Statistical analysis

GraphPad Prism (GraphPad Software, La Jolla, CA) was used for data analysis (Student’s t-test).

## Results

### Endothelial permeability is increased after irradiation

**Fig. 1:**
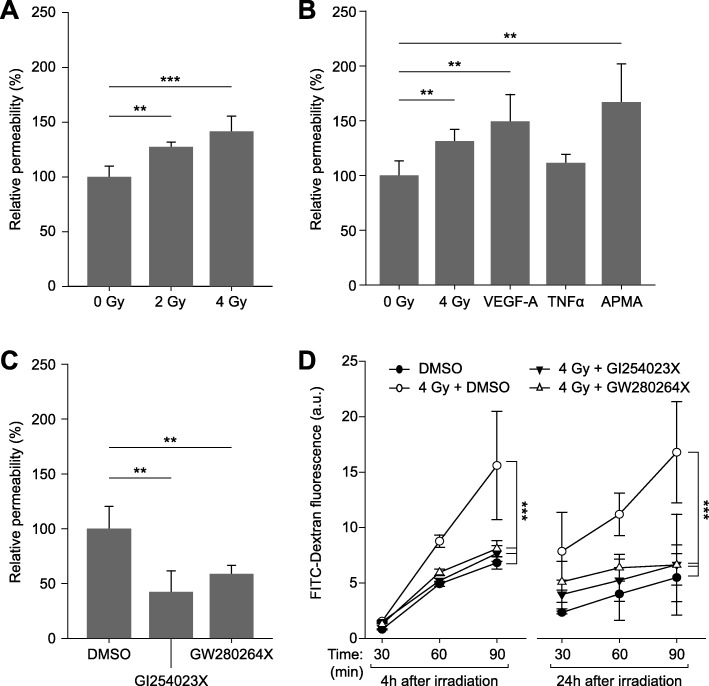
Endothelial cell monolayer permeability assays using FITC-dextran. **a**) Relative permeability 4 h after irradiation, compared to non-irradiated controls (0 Gy). **b**) Relative permeability of cell monolayers measured 24 h after irradiation with 4 Gy, after treatment with VEGF-A (100 ng/ml) or TNFα (100 ng/ml) for 24 h, and after exposure to APMA (10 ng/ml) for 2 h, compared to vehicle (DMSO, 0.1%) only-treated controls. **c**) Effects of ADAM inhibitors GI254023X (10 μM; specific for ADAM10 only) and GW280264X (10 μM; inhibits both ADAM10 and ADAM17). Inhibitor or vehicle were added to the monolayers 24 h before measurement. **d)** ADAM inhibitors counteract the irradiation-induced increase in permeability. Measurements were performed 24 h after addition of inhibitors and 4 h (left) or 24 h (right) after irradiation, respectively. Data shown are means (*n* ≥ 3) and standard deviations. Statistics: t-test, **p < 0.01, ***p < 0.001

The effect of ionizing radiation on the permeability of an endothelial monolayer was investigated and compared with the effects of known permeability-inducing agents such as VEGF (vascular endothelial growth factor-A) [14], TNFα (tumor necrosis factor alpha [15], as well as of APMA (4-aminophenylmercuric acetate) [16], an activator of matrix metalloproteinases. Irradiation with photons significantly and dose-dependently increased the permeability of endothelial cell monolayers by 25% at 2 Gy and by 35% at 4 Gy when compared to non-irradiated controls (Fig. 1a). This increase was comparable to that achieved by permeability-increasing substances (Fig. 1b).

### ADAM inhibitors counteract the radiation-induced increase in endothelial permeability

Treating endothelial cell monolayers with the ADAM10 inhibitors GI254023X and GW280264X (also inhibiting ADAM17) led to reduced permeability corresponding to approx. 40 and 60%, respectively, of that of controls treated with vehicle (DMSO) alone (100%; Fig. 1c). Both inhibitors also reduced the radiation-induced increase in the permeability of endothelial cell monolayers (Fig. 1d).

### Expression and activation of ADAM10, but not of ADAM17, is increased in irradiated endothelial cells

**Fig. 2:**
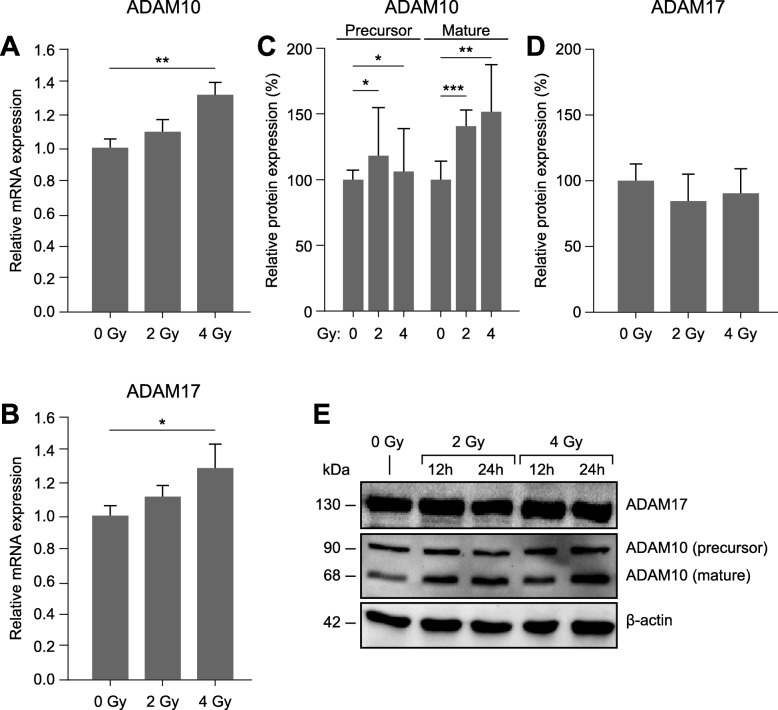
Effect of ionizing radiation on the expression levels of ADAM10 and ADAM17 in endothelial cells. **a** and **b**) ADAM10 (A) and ADAM17 (B) mRNA levels 24 h after irradiation with 2 Gy or 4 Gy, relative to those in non-irradiated controls (ΔΔCT-method). **c**-**d**) Quantitative immunoblot analysis. ADAM10 (C) and ADAM17 (D) protein levels (normalized to β-actin) measured 24 h after irradiation are shown relative to those in non-irradiated controls. **e**) Exemplary immunoblot showing protein bands 12 h and 24 h after irradiation. Values shown are means (n ≥ 3) and standard deviations. Statistics: t-test, *p < 0.05, **p < 0.01, *p < 0.001

The lack of irradiation-induced permeability increases in the presence of ADAM inhibitors implicated these proteases as possible mediators of this effect. Therefore, we wanted to know whether the expression levels of ADAM10 and ADAM17 were influenced by irradiation. While both, ADAM10 (Fig. 2a) and ADAM17 (Fig. 2b) were upregulated on the mRNA level, only ADAM10 protein levels, especially those of its mature (i.e. active) form (68-kDa-fragment) were increased (Fig. 2c and e). ADAM 17 protein levels remained constant (Fig. 2d and e).

### Irradiation of endothelial cells leads to degradation of VE-cadherin

**Fig. 3:**
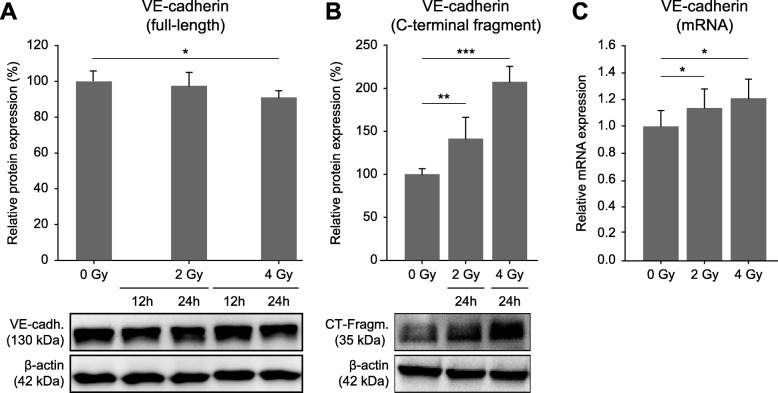
Influence of ionizing radiation on the expression of VE-cadherin in endothelial cells. **a**) Quantitative immunoblot analysis of VE-cadherin expression 24 h after irradiation (*n* = 4). Data were normalized to β-actin levels and are shown relative to the non-irradiated control (0 Gy). **b**) Quantitative immunoblot analysis of a 35-kDa proteolytic VE-cadherin fragment 24 h after irradiation (C, *n* = 3; data as described in **a**). **c**) Quantification of VE-cadherin mRNA expression levels 24 h after irradiation (n = 3; ΔΔCT method with GAPDH as reference target; data is shown relative to the non-irradiated control). Exemplary immunoblots are shown in A and B. Data shown are means ± standard deviations. Statistics: t-test, *p < 0.05, **p < 0.01, ***p < 0.001

VE-cadherin is a known target of ADAM10 proteolysis [13] and is an important component of adherens junctions, contributing to endothelial permeability [7, 8]. Therefore, we were interested to see whether exposure to ionizing radiation affected the level of VE-cadherin expression. Immunoblot analyses of lysates prepared from endothelial cell monolayers 12 h and 24 h after irradiation showed decreasing VE-cadherin (Fig. 3a). This effect was more pronounced after 24 h and appeared to be due to increased degradation, as the levels of a 35-kDa proteolytic fragment increased in an irradiation dose-dependent manner, up to > 2-fold compared to non-irradiated controls (Fig. 3b). On the transcript level, we detected up to about 1.2-fold higher mRNA expression 24 h after irradiation (Fig. 3c).

### Inhibition of ADAM10 stabilizes VE-cadherin and prevents its irradiation-induced degradation

**Fig. 4:**
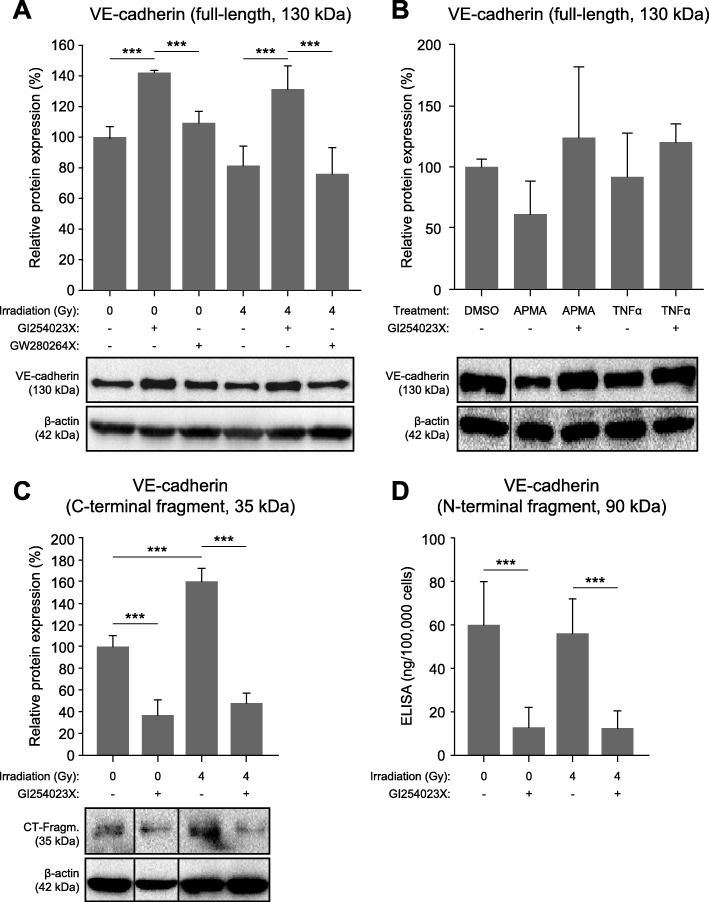
Effect of ADAM inhibitors on VE-cadherin protein levels. **a**) Endothelial cells pre-treated 30 min before irradiation (4 Gy) with vehicle alone (DMSO, 0.1%) or with inhibitors of ADAM10 (GI254023X, 10 μM), and ADAM17 (GW280264X, 10 μM) were lyzed and subjected to immunoblot analysis and quantitative evaluation (n ≥ 3; β-actin served as loading control). **b**) Endothelial cells were in the presence of absence of the ADAM10-inihibitor GI254023X (10 μM) treated with APMA (100 ng/ml; for 2 h only) or TNFα (100 ng/ml) and analyzed 24 h later as described in A (*n* ≥ 2). **c**) Quantification of the 35-kDa intracellular C-terminal fragment of VE-cadherin detected by immunoblot analysis as described in A but in the presence of a γ-secretase-I inhibitor (1 μM) in order to stabilize the proteolytic fragment (n ≥ 3). **d**) Quantification of the soluble 90-kDa N-terminal VE-cadherin fragment by ELISA. For this purpose, a total of 10^6^ cells in 3 ml medium were seeded into 8-cm^2^-dishes 24 h before and treated with GI254023X (10 μM) 30 min before irradiation (4 Gy). After 24 h, the cell culture supernatant was assayed and the amount of soluble VE-cadherin (ng) per 100,000 cells originally seeded was calculated (*n* ≥ 4). Exemplary immunoblots are shown (**a**–**c**). Data are shown as means ± standard deviations. Statistics: t-test, *p < 0.05, **p < 0.01, ***p < 0.001

To further test the hypothesis that irradiation-induced degradation of VE-cadherin is mediated by ADAM10, we measured VE-cadherin protein levels in endothelial cells pre-treated with the ADAM inhibitor GI254023X or GW280264X (Fig. 4a). In the presence of the ADAM10-specific inhibitor, VE-cadherin was stabilized at considerably higher levels compared to control cells, both in non-irradiated cells as well as in endothelial cells irradiated with a dose of 4 Gy. This effect was not observed with GW280264X. Interestingly, both GI254023X and GW280264X led to a reduction to about 50% or the mature form (68 kDa) of the ADAM10 protease, while the levels of its precursor (90 kDa) or ADAM17 were not affected (data not shown). The protease activator APMA [16] and TNFα [15] are both known to lead to increased degradation of VE-cadherin. In the presence of the ADAM10-specific inhibitor GI254023X, this effect was also blocked (Fig. 4b). Next, we investigated the degradation of VE-cadherin in more detail by analyzing both resulting fragments, the 35-kDa C-terminal intracellular fragment (immunoblot, Fig. 4c) and the soluble 90-kDa N-terminal extracellular fragment (ELISA, Fig. 4d). Irradiation increased the cleavage of VE-cadherin and correspondingly led to increased detection of the 35-kDa fragment. However, a corresponding increase in the amount the soluble fragment was not observed. In the presence of the ADAM10-specific inhibitor GI254023X, levels of both proteolytic fragments were decreased to similarly low levels (about 40 and 20%, respectively), irrespective of irradiation.

### In addition to degradation, irradiation leads to dislocalization of VE-cadherin in endothelial cell layers

**Fig. 5:**
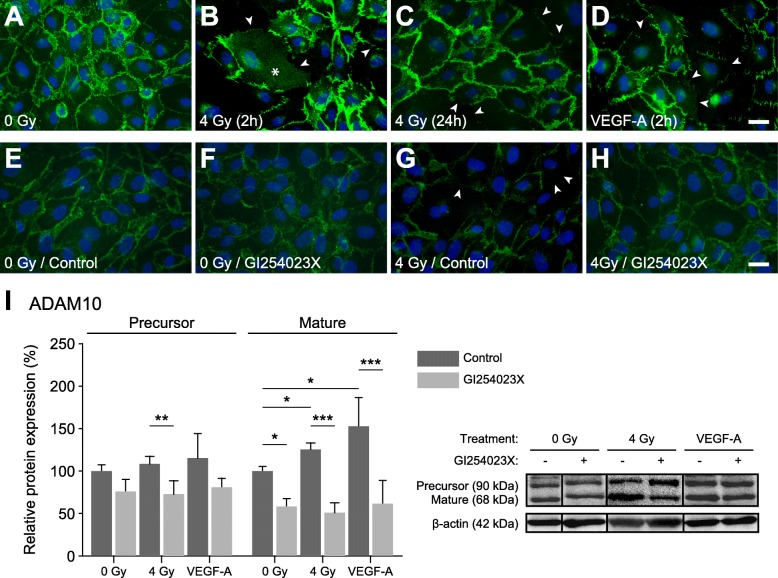
Irradiation-induced dislocalization and degradation of VE-cadherin and VEGF-A-induced activation of ADAM10. **a**–**d**) Immunofluorescence stainings showing subcellular distribution of VE-cadherin in endothelial cells grown on coverslips. Upon reaching confluence, cells were mock-irradiated (**a**), irradiated with 4 Gy (**b** and **C**), or treated with 100 ng/ml VEGF-A (**d**) and prepared for VE-cadherin (green; Hoechst-33,342 nuclear staining is shown in blue) immunofluorescence microscopy after 2 h (B and D) or 24 h (C; 4 Gy only). Arrowheads indicate weakened or absent VE-cadherin staining at cell-cell contact sites. Asterisks mark areas of granular VE-cadherin staining indicating dislocation from cell-cell contact sites. **E–H**) VE-cadherin localization in control and 4 Gy-irradiated endothelial cell layers in the absence or presence of the ADAM10-inhibitor GI254023X (10 μM). Cells were fixed and stained for VE-cadherin (green; nuclei are blue) after 24 h. Scale bars in A–H, 20 μm. **I**) ADAM10 expression (precursor and mature form) in endothelial cells treated with irradiation (4 Gy; proteins isolated after 24 h) or VEGF-A (100 ng/ml; proteins isolated after 4 h) in the absence or presence of GI253023X (10 μM; added 30 min before treatments). Data (n ≥ 3) are shown as means ± standard deviations. Statistics: t-test, *p < 0.05, **p < 0.01, ***p < 0.001

As mentioned above, in contrast to the small intracellular C-terminal VE-cadherin fragment that results from proteolytic cleavage, the soluble 90-kDa extracellular fragment did not show the expected parallel increase after irradiation. Therefore, we used immunofluorescence microscopy to analyze the localization of VE-cadherin in endothelial cell layers after irradiation. For comparison, we also treated cells with recombinant VEGF-A, which is known to induce accelerated endocytosis of VE-cadherin and thus disturb the endothelial barrier [17]. While control cells showed strong expression of VE-cadherin and clear localization at cell-cell contact sites (Fig. 5a), irradiated cells (4 Gy) or cells treated with recombinant VEGF-A, after 2 h, showed a clear reduction of VE-cadherin staining at cell-cell contact sites (arrowheads, Fig. 5b and d, respectively). In case of irradiation, in addition to being reduced, VE-cadherin appeared to be dislocalized to a higher degree than after VEGF-A treatment (granular staining marked by asterisks in Fig. 5b), but this effect was transient, as after 24 h, while VE-cadherin was still reduced at cell-cell contact sites, the granular staining was comparable to that in control cells (Fig. 5c). In the presence of the ADAM10 inhibitor GI254023X, irradiation did not induce reduction or dislocalization of VE-cadherin (Fig. 5e–h). When we looked at ADAM10 expression, we found that both, irradiation and VEGF-A, increased expression of ADAM10 and specifically its mature form, and that this was effectively blocked by GI254023X (Fig. 5i). These results and that VEGF was shown to mediate permeability of the endothelium via ADAM10-induced degradation of VE-cadherin [18], led us to ask whether the effects observed after irradiation might be due to an induction of VEGF-A expression in endothelial cells, but no differences in VEGF-A (measured by ELISA) were detected in cell culture supernatants of irradiated and non-irradiated endothelial cells (data not shown).

### ADAM10-inhibition prevents increased transendothelial migration of tumor cells after irradiation

**Fig. 6:**
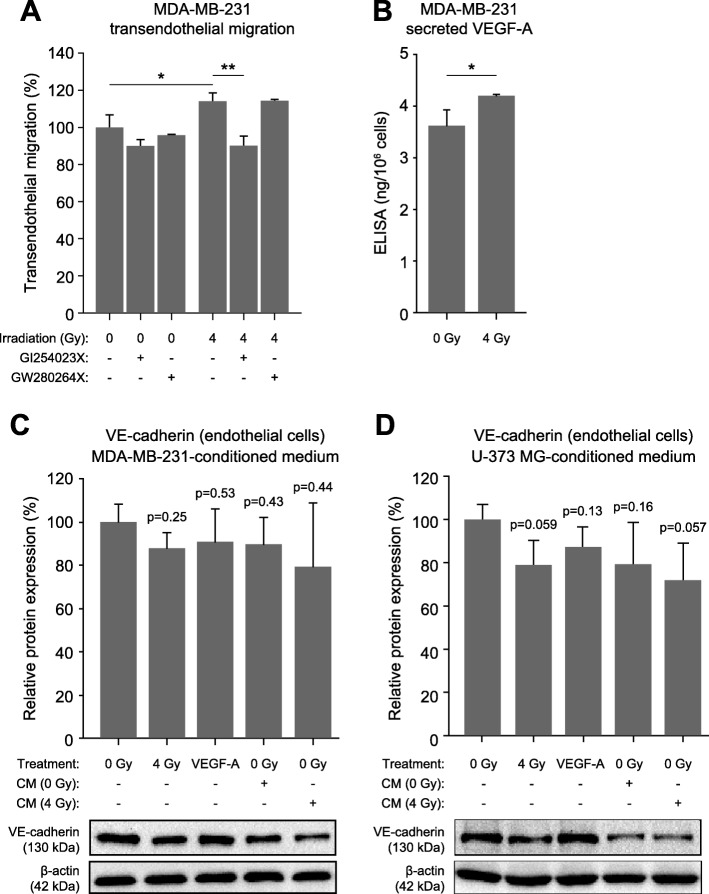
MDA-MB-231 transendothelial migration and VEGF-A production. **a**) Transendothelial cell migration assay showing the effect of endothelial cell irradiation (4 Gy) in the absence or presence of ADAM10/17 inhibitors on the transmigration of MDA-MB-231 breast tumor cells (n ≥ 3). **b**) VEGF-A content in MDA-MB-231 cell culture supernatants measured by ELISA 24 h after irradiation (mock or 4 Gy; n ≥ 3). **c** and **d**) Immunoblot analysis of VE-cadherin expression after irradiation (4 Gy), after treatment with recombinant VEGF-A (100 ng/ml), and after treatment with conditioned medium (CM; harvested after 24 h) from non-irradiated or irradiated (4 Gy) MDA-MB-231 cells (C; *n* = 2) and U-373 MG cells (D; n = 3) (lysates prepared after 24 h or 2 h in case of VEGF-A treatment). Data are absolute values (**b**) or relative to those of controls (**a**, **c**) and shown as means ± standard deviations. Statistics: t-test, *p < 0.05, **p < 0.01

Irradiation of endothelial cell monolayers increases their permeability also for tumor cells, as demonstrated in the case of the breast cancer cell line MDA-MB-231 (Fig. 6a). Transendothelial tumor cell migration was reduced by about 10% and the irradiation-induced permeability increase was completely blocked in the presence of the ADAM10-specific inhibitor GI254023X, but not GW28064X (Fig. 6a).

### Tumor cell-secreted VEGF-A contributes to the degradation of VE-cadherin in endothelial cells

Since most tumors produce VEGF-A, we wanted to assess whether irradiation increased VEGF-A production in tumor cells and what the effect of this on VE-cadherin levels in endothelial cells was. To this end, we irradiated MDA-MB-231 cells with 4 Gy and measured the VEGF-A content in the cell culture supernatant after 24 h by ELISA (Fig. 6b), which led to an approx.15% increase in VEGF-A. Next, we exposed endothelial cell layers to conditioned medium from non-irradiated and irradiated tumor cell cultures and determined expression levels of VE-cadherin after 24 h by quantitative immunoblot analysis (Fig. 6c, d). Conditioned medium from non-irradiated MDA-MB-321 led to a reduction in VE-cadherin levels comparable to that observed when endothelial cells were irradiated or treated with recombinant VEGF-A. Conditioned medium from irradiated MDA-MB-231 led to an even further decrease in VE-cadherin levels (Fig. 6c). These results were confirmed in experiments using the glioblastoma cell line U-373 MG cell line (Fig. 6d).

## Discussion

Radiotherapy, alone or in combination with chemotherapy, is used with great success in neoadjuvant and adjuvant settings. However, despite enormous medical progress in the treatment of tumors, recurrences or metastases occur in most cases. Here, we investigated the effects of ionizing radiation on endothelial cell monolayers and how changes in their molecular composition and integrity affected their interaction with tumor cells. We found that photon-irradiation of endothelial monolayers with therapeutic doses led to increased endothelial permeability and transmigration of tumor cells. Specifically, we found that, upon irradiation, the metalloprotease ADAM10 underwent a shift from its precursor to the mature form, resulting in increased degradation and dislocalization of VE-cadherin, one of the main constituents of endothelial cell contact sites and vital for their integrity, maintenance and regulation. We showed that these irradiation-induced effects are similar to those induced by VEGF-A or by the protease-activator APMA, and that they could be inhibited by ADAM10 (but not ADAM17)-specific inhibitors. However, we could rule out VEGF-A as a mediator of these irradiation-induced effects. On the other hand, we found that tumor cells, such as MDA-MB-231, secreted higher levels of VEGF-A after irradiation, and that this contributed to the degradation of endothelial integrity through cleavage of VE-cadherin.

The notion that irradiation increases endothelial permeability is not new. Hamalukic et al., for instance, reported increased extravasation and subsequent metastasis of intravenously injected tumor cells after whole-body irradiation of naked mice [19]. While these authors attributed this on increased expression of several types of adhesion molecules in both, endothelial and tumor cells, in turn leading to increased tumor cell – endothelial cell adhesion and subsequent extravasation of tumor cells, we show here that through the degradation (mediated by ADAM10) and dislocalization of VE-cadherin, irradiation compromises the endothelial barrier function directly. This likely contributed to the effect observed in mice.

Recently, this mechanism of ADAM10-mediated breakdown of VE-cadherin upon exposure to ionizing radiation, leading to increased endothelial permeability, has been described by Kabacik and Raj in the context of increased risk of cardiovascular diseases after radiotherapy [20]. Here, the authors proposed that irradiation leads to the production of reactive oxygen species that in turn cause an increase in intracellular Ca^2+^ concentrations leading to ADAM10-activation. Our results are in agreement with these data, showing that these consequences of irradiation already manifest very shortly, within 2 h, but are persistent (24 h in our experiments; Kabacik and Raj performed most of their analyses 7 days after irradiation). Furthermore, we can exclude any relevant involvement of ADAM17 and confirm the VEGF-independence of this mechanism. In our permeability assays, we had found that ADAM10 as well as ADAM17-inhibitors prevented an irradiation-induced increase in permeability of endothelial cell monolayer for macromolecules, but only the ADAM10 inhibitor was able to counteract VE-cadherin cleavage and transendothelial migration of MDA-MB-231 breast cancer cells. This confirms that ADAM17 is not directly involved in the regulation of VE-cadherin-mediated permeability. This limited permeability-decreasing effect of the ADAM17 inhibitor could be explained by it preventing the activation of ADAM17 substrates such as, for example, TNFα, which has been described to increase permeability [21]. Additionally, ADAM10 and ADAM17 cleave further adhesion molecules such as JAM-A *(junctional adhesion molecule A*) and thereby regulate transendothelial leukocyte migration and ADAM17 was thought to be the main mediator of this cleavage [22]. On the other hand, Flemming et al. measured an increase in vascular permeability induced by lipopolysaccharides (LPS) and TNFα, which was associated with an increased cleavage and release of soluble VE-cadherin [23]. In our assays, TNFα only led to a marginal increase in permeability (not statistically significant), while the effect of irradiation was comparable to that of VEGF-A [14] and APMA [16], substances known to increase endothelial permeability.

With our data, we can neither confirm nor refute the mechanism of ADAM10 activation proposed by Kabacik and Raj [20], but it is quite possible that some upstream enzymes are activated that then induce the activation of ADAM10. Lee et al., for instance, reported a correlation between the increase in expression of the enzyme furin in tumor cells and in samples from patients with laryngeal tumor after irradiation, with an increased expression of the active form of metalloproteinase MMP-2 [24]. It is known that most metalloproteinases, including ADAM10, are activated by furin-like enzymes or convertases [25].

Interestingly, we noted that while we could detect proportional levels of the C-terminal fragment with the proteolytic degradation of VE-cadherin, this was not the case with its soluble N-terminal fragment. Immunofluorescence microscopy revealed that in addition to the cleavage and loss of VE-cadherin at endothelial cell junctions, VE-cadherin was shifted, presumably by internalization, to other compartments inside the cells. It is therefore possible that ionizing radiation affects the permeability of the endothelium not only through cleavage of VE-cadherin by ADAM10, but additionally by dislocalization of this protein. Several studies have already reported on the regulation of endothelial permeability via internalization of VE-cadherin. For example, Gavard et al. showed that a 30-min treatment of endothelial cells with recombinant VEGF led to a reversible internalization of VE-cadherin [17]. Notably, the irradiation-induced downregulation and dislocalization of VE-cadherin differed from that induced by treatment with recombinant VEGF-A. In the former case, after 2 h, there was noticeable more dislocalized VE-cadherin while the reduction at cell-cell contact sites was comparable. After 24 h, the granular VE-cadherin staining was no longer apparent in irradiated cells, while staining at cellular junctions was still reduced. Thus, internalization appears to be a short-term effect of irradiation. This further supports the finding that the effects induced by irradiation are mechanistically independent of the VEGF pathways.

Finally, when we looked at tumor cells and their interaction with endothelial cell monolayers, we found increased transendothelial migration of MDA-MB-231 cells through irradiated endothelia that could be reduced to baseline levels when inhibiting ADAM10. Furthermore, upon irradiation of tumor cells, their production of VEGF-A was increased from baseline levels, similar to what has been described by others for e.g. glioma cells [26]. Exposure of endothelial cell monolayers to conditioned medium from non-irradiated MDA-MB-231 cells led to degradation of VE-cadherin to an extent similar to irradiation of monolayers or treatment with recombinant VEGF-A, and irradiation of tumor cells had an additive effect. This suggests that VEGF released by tumor cells contributes to VE-cadherin degradation. In the irradiated setting, such as after localized radiotherapy, these effects are likely compounded, facilitating transendothelial migration of tumor cells, i.e. intravasation and extravasation, crucial steps of metastasis.

## Conclusion

In summary, our data show that ionizing irradiation can activate the metalloproteinase ADAM10 in endothelial cells and thereby increase the vascular permeability through degradation and dislocalization of VE-cadherin, which facilitates transendothelial migration of tumor cells. Furthermore, irradiation of tumor cells can lead to increased secretion of factors such as VEGF-A, which further add to the weakening of the endothelial barrier.

## Data Availability

The datasets used and/or analyzed during the current study are available from the corresponding author at reasonable request.

## Abbreviations

ADAM: a disintegrin and metalloproteinase
APMA: 4-Aminophenylmercuric acetate
HUVEC: Human umbilical vein endothelial cells
JAM-A: junctional adhesion molecule A
LPS: lipopolysaccharides
TNFα: Tumor necrosis factor alpha
VE-cadherin: Vascular endothelial cadherin
VEGF-A: Vascular endothelial growth factor-A
